# Hypoxic mesenchymal stem cell-derived exosomes promote the survival of skin flaps after ischaemia–reperfusion injury via mTOR/ULK1/FUNDC1 pathways

**DOI:** 10.1186/s12951-023-02098-5

**Published:** 2023-09-21

**Authors:** Chao Deng, Kangkang Dong, Yongjun Liu, Ken Chen, Chuwei Min, Zheming Cao, Panfeng Wu, Gaojie Luo, Gechang Cheng, Liming Qing, Juyu Tang

**Affiliations:** 1 Department of Orthopedics, Xiangya Hospital, Central South University, Changsha, China; 2 National Clinical Research Center for Geriatric Disorders, Xiangya Hospital of Central South University, Changsha, China

**Keywords:** Exosomes, Hypoxia, Bone marrow mesenchymal stem cells, Ischaemia–reperfusion, Skin flap, miRNA, Autophagy, 3-MA

## Abstract

Flap necrosis, the most prevalent postoperative complication of reconstructive surgery, is significantly associated with ischaemia–reperfusion injury. Recent research indicates that exosomes derived from bone marrow mesenchymal stem cells (BMSCs) hold potential therapeutic applications in several diseases. Traditionally, BMSCs are cultured under normoxic conditions, a setting that diverges from their physiological hypoxic environment in vivo. Consequently, we propose a method involving the hypoxic preconditioning of BMSCs, aimed at exploring the function and the specific mechanisms of their exosomes in ischaemia–reperfusion skin flaps. This study constructed a 3 × 6 cm^2^ caudal superficial epigastric skin flap model and subjected it to ischaemic conditions for 6 h. Our findings reveal that exosomes from hypoxia-pretreated BMSCs significantly promoted flap survival, decrease MCP-1, IL-1β, and IL-6 levels in ischaemia–reperfusion injured flap, and reduce oxidative stress injury and apoptosis. Moreover, results indicated that Hypo-Exo provides protection to vascular endothelial cells from ischaemia–reperfusion injury both in vivo and in vitro. Through high-throughput sequencing and bioinformatics analysis, we further compared the differential miRNA expression profiles between Hypo-Exo and normoxic exosomes. Results display the enrichment of several pathways, including autophagy and mTOR. We have also elucidated a mechanism wherein Hypo-Exo promotes the survival of ischaemia–reperfusion injured flaps. This mechanism involves carrying large amounts of miR-421-3p, which target and regulate mTOR, thereby upregulating the expression of phosphorylated ULK1 and FUNDC1, and subsequently further activating autophagy. In summary, hypoxic preconditioning constitutes an effective and promising method for optimizing the therapeutic effects of BMSC-derived exosomes in the treatment of flap ischaemia–reperfusion injury.

## Introduction

Flap transplantation represents a commonly utilized procedure within the field of plastic and reconstructive surgery [1]. Despite significant improvements in the survival rates of skin flap transplants [2], the incidence of partial flap necrosis remains relatively high, at approximately 7–20% [3–5], A key contributing factor to skin flap necrosis is ischaemia–reperfusion (I/R) injury, associated with reactive oxygen species generation, inflammation, cellular necrosis, and apoptosis [6]. This sequence of pathophysiological events negatively impacts the viability of flaps, thus reducing their overall survival rates. Accordingly, several therapeutic strategies have been proposed to mitigate I/R injury, encompassing drug-based therapies such as melatonin [7], febuxostat [8], and cell-based therapies including the use of adipose stem cells [9]. While these methods have exhibited some efficacy in animal models, their clinical effectiveness remains to be confirmed.

Exosomes, a subtype of extracellular vesicles, originate from the inward budding of intracellular lysosomal particles, giving rise to multivesicular bodies. The fusion of the outer membranes of these multivesicular bodies with the cell membrane ultimately leads to the production of exosomes. These vesicles, typically measuring between 30 and 200 nm in diameter, are known to transport an array of biological compounds such as cytokines, mRNAs, miRNAs, and proteins [10]. Through the binding of surface ligands to target cells and the delivery of their internal contents, exosomes regulate a multitude of biological processes, often mirroring the functional attributes of their cells of origin. Currently, exosomes derived from mesenchymal stem cells have demonstrated therapeutic potential in several diseases [11–13], most notably in the context of ischaemia–reperfusion injury [14–16]. Intriguingly, exosomes derived from hypoxia-preconditioned BMSCs may carry altered cargos, including varied miRNAs, thus significantly enhancing their biological functionality [17–19]. Previous research has suggested that exosomes from hypoxia-preconditioned mesenchymal stem cells can promote fracture healing [17]. Despite these advancements, their specific role and underlying mechanisms in ischaemia–reperfusion injuries within flaps remain to be elucidated.

Autophagy is a critical biological process within cells, encompassing the degradation and recycling of cellular waste and damaged organelles. In ischaemia–reperfusion injury (IR), the role of autophagy in various organs may present a double-edged sword characteristic, exerting both protective and potentially damaging effects [20]. For instance, moderate autophagy could be beneficial for cardiomyocyte survival in cardiac IR, while excessive autophagy might exacerbate myocardial damage [21]. Similar conclusions have been drawn from cerebral IR models, where autophagy displays complex effects on neuronal cell death and neurological function recovery. Appropriate autophagy might protect neurons, but excessive autophagy could result in neuronal death [22]. Meanwhile, in liver IR studies, the activation of autophagy might help mitigate cellular damage, protecting the liver by clearing damaged mitochondria and suppressing inflammatory responses [23]. Autophagy also plays a crucial role in kidneys and the intestine, acting as a key player in the protection of renal tubular epithelial cells [24], while aiding in the alleviation of intestinal mucosal damage [25].

In recent years, researchers have discovered that miRNAs carried in exosomes play a significant role in regulating autophagy. miRNAs are short non-coding RNAs that control gene expression by binding to the 3’UTR of target genes, causing mRNA degradation or translation suppression. Within exosomes, miRNAs can be encapsulated and transferred between cells via exosomes, thereby influencing the biological functions of target cells. Existing studies have confirmed that miRNAs in exosomes can target autophagy-related genes, such as mTOR, ULK1, Beclin 1, ATG5, ATG7, etc., regulating aspects of the autophagic process including initiation, vesicle formation, and fusion [26].

This study employed hypoxic preconditioning of bone marrow mesenchymal stem cells (BMSCs) and collection of their exosomes to investigate the effects of Hypo-Exo on ischaemia–reperfusion flaps and unravel the underlying mechanisms.

## Results

### Biological function of Hypo-Exo

**Fig. 1:**
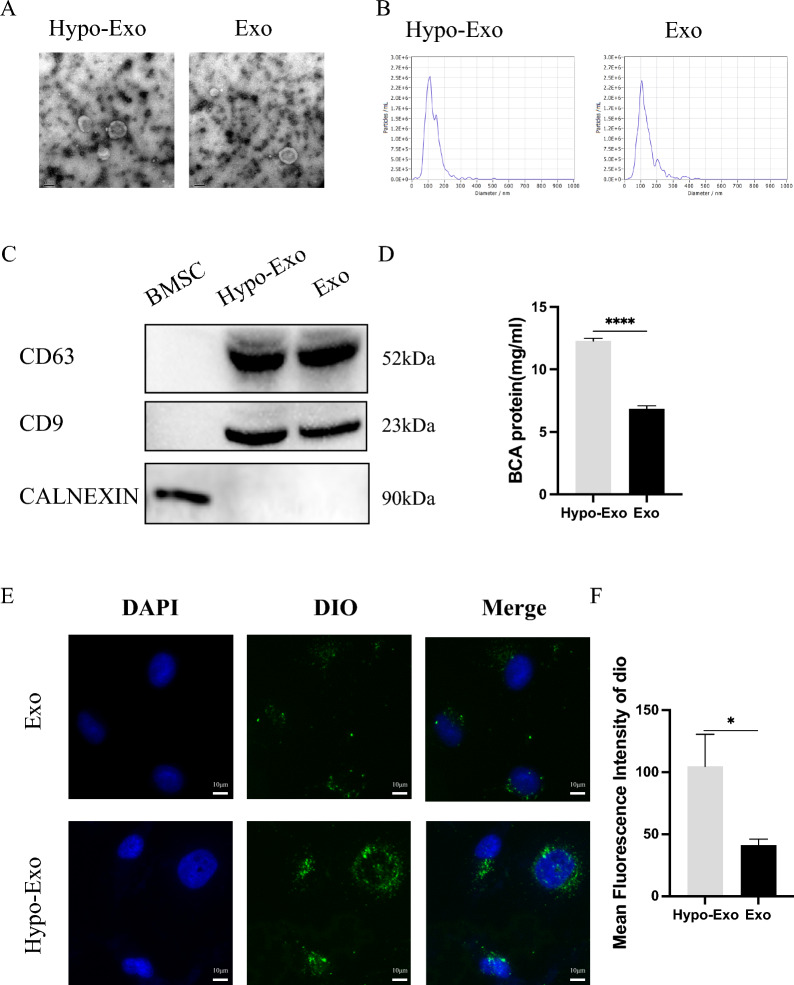
Characterization of Exosomes. **A** Morphology of Hypo-Exo and Exo under TEM. **B** NTA particle analysis of Hypo-Exo and Exo, two groups share similar size ranges (40–150 nm). **C** Western blotting analysis showing the the expression of CD63, CD9 and CALENXIN. **D** The BCA assay was used to measure the exosome protein concentration in the two groups (****P < 0.0001). **E** Uptake of the green fluorescence dye Dio labelled Hypo-Exo and Exo into HUVECs (bar size 10 μm). **F** Statistical evaluation of Mean fluorescence intensities in the two groups (*P < 0.05). Bars indicated means ± SD

The potential biological functions of Hypo-Exo were initially explored. Both Exo and Hypo-Exo were identified as per the guidelines established by previous research [27, 28]. TEM results revealed that both Hypo-Exo and Exo had a diameter of approximately 100 nm (Fig. 1A), with NTA further demonstrating a similar size distribution (Hypo-Exo vs. Exo: average 115.3 nm vs. 117.3 nm) (Fig. 1B). Concurrently, western blot results indicated a high expression of exosomal surface markers (CD9 and CD63) in both Hypo-Exo and Exo (Fig. 1C). These characterization studies confirmed that Hypo-Exo and Exo share identical morphological characteristics, including size, shape, and biomarkers. Notably, it was discovered that hypoxia could enhance exosome release from BMSCs. BCA assays indicated that total protein concentrations of exosomes derived from BMSCs (subjected to 3% O^2^ for 24 h) were significantly higher compared to those exposed to normal O^2^ levels (Fig. 1D).

In addition, the role of exosomes in modulating distant and target cells was assessed by measuring their uptake rate. To ascertain the quantity of exosomes endocytosed by HUVECs, the average fluorescence intensity of the cells was computed. It was demonstrated that Dio-labeled Hypo-Exo were more readily endocytosed by HUVECs following 6 h of co-culture (Fig. 1E, F).

### Hypo-Exo improved the survival rate of I/R injured skin flaps through attenuating I/R injury

**Fig. 2:**
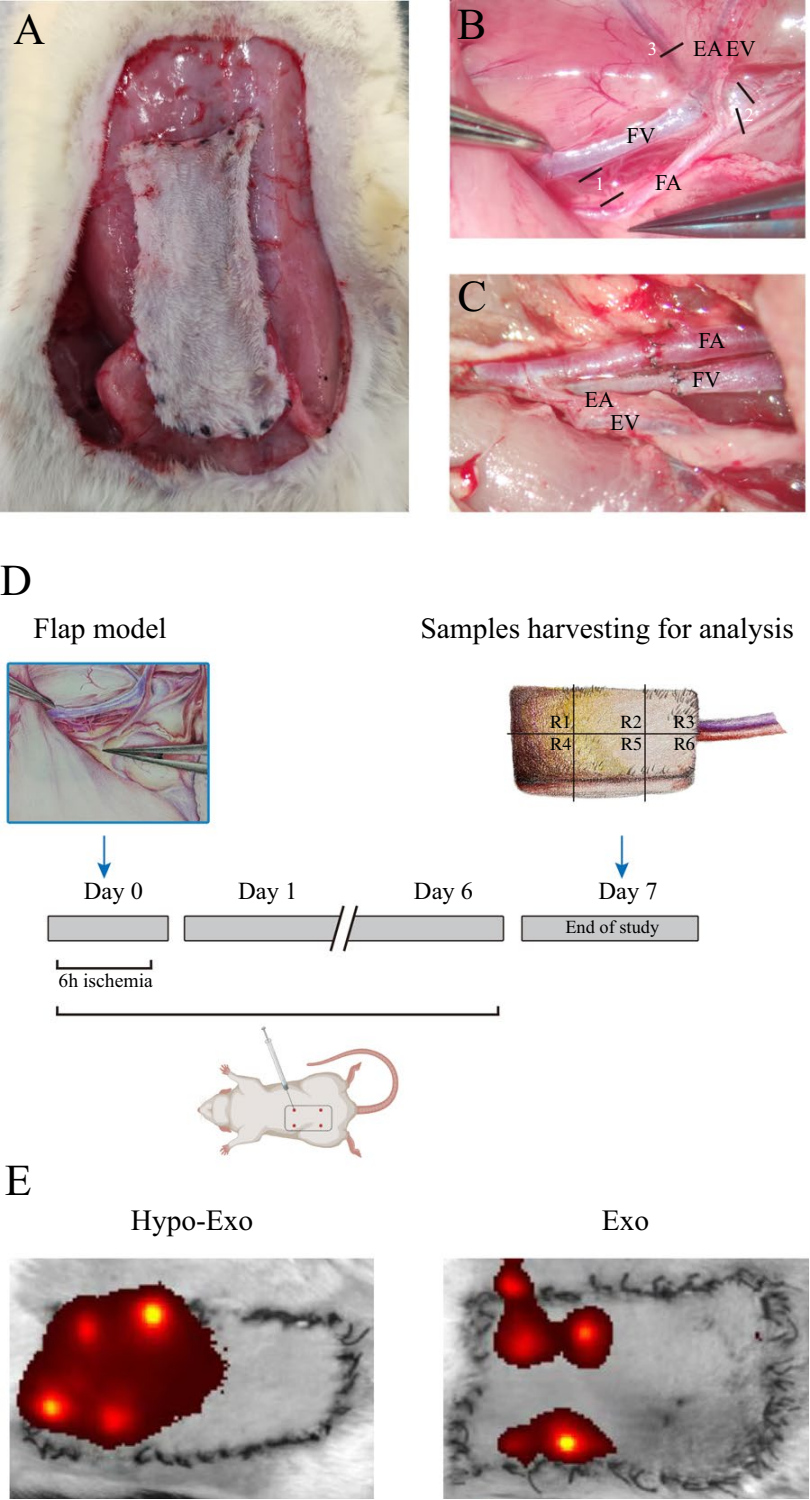
Flap model, Schematic diagram of surgery and experimental design. **A** The caudal superficial epigastric skin free flap (3 × 6 cm^2^). **B** Intraoperative Vascular Anatomy: *EA* epigastric artery, *EV* epigastric vein, *FA* femoral artery, *FV* femoral vein, 1 (ligatures of lateral circumflex FA and FV), 2 (ligatures of saphenous artery and vein), 3 (ligatures of proximal caudal FA and FV). **C** Anastomose the femoral artery and femoral vein with 10–0 nylon suture. **D** Experimental process design. **E** In vivo tracking of DiR-labeled exosomes

This study successfully established a rat flap ischaemia–reperfusion model (Fig. 2A, B, C). As described in the methodology, the femoral artery and vein were sutured under a microscope without iatrogenic vascular damage, and the rat flap had good blood supply post-operation, appearing warm and reddish. Two hours after the operation, a medical cotton swab was used to press on the flap for 1 s before release, and all flaps resumed their normal color within 3–5 s. At the study's observation endpoint, no rat flap blood flow was affected by anatomical separation, vascular ligation, or anastomosis, and no limp was observed in any rat. On the day of surgery, DIR fluorescence-labelled Hypo-Exo and Exo were subcutaneously injected into the rat flaps in the Hypo-Exo and Exo groups. Upon re-examination after 24 h, it was found that Hypo-Exo diffused more evenly within the flaps compared to Exo, exhibiting higher fluorescence intensity (Fig. 2E).

**Fig. 3:**
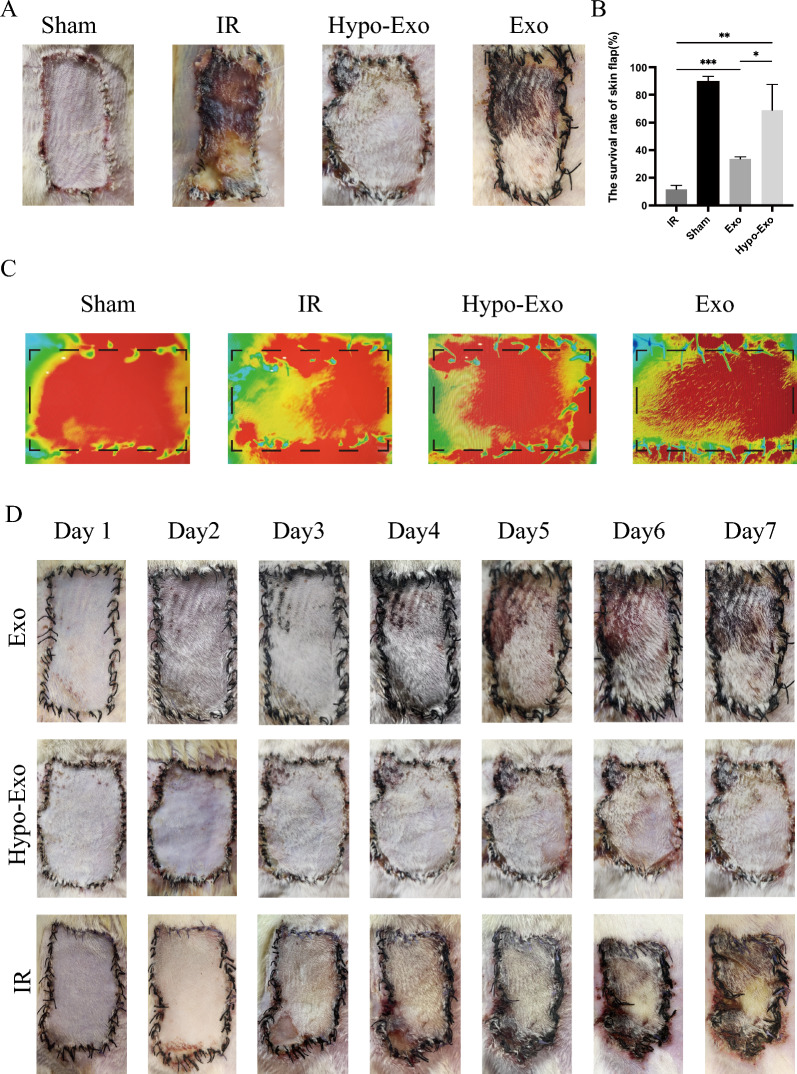
Flap appearance and survival rate. **A** The skin flap appearance of each group on the 7th day after operation. **B** The flap survival rate of each group on the 7th day after operation (*P < 0.05, **P < 0.01, ***P < 0.001). **C** Angiography images of each group on the 7th day after operation. **D** Changes of skin flap appearance in each group from the 1st day to the 7th day after operation. Bars indicated means ± SD

To explore whether Hypo-Exo treatment is beneficial for flap survival after I/R injury, we examined the morphological changes in the skin as well as the flap survival rate. Generally, in the Sham group, only the edge of the skin at the suture line was slightly red and swollen in the first few days. Over time, the redness and swelling of the skin gradually subsided, and on the 7th day, almost all of the flaps survived, and the flaps were soft with good hair growth on the surface (Fig. 3A). In contrast, in the I/R group, after exposure to ischaemia–reperfusion, the flaps appeared to be blue‒purple in colour, with swelling and exudation. After 24 h, the superficial swelling increased. On 3rd day, the swelling gradually subsided, but inflammatory exudate was observed at the suture. On 4th day, necrotic scabs began to appear in the flaps, and then, the area of necrosis gradually increased, some flaps became leathery or sclerotic, and there was no obvious hair growth (Fig. 3D). In addition, on the 7th day, the blood flow signal intensity in the necrotic area was significantly weakened in the I/R group (Fig. 3C). The Hypo-Exo group performed similarly to the I/R group in the early stages of ischaemia–reperfusion. However, in the Hypo-Exo group, the swelling had significantly subsided on the 3rd day, and the color of the flap returned to normal. Similarly, necrosis began to appear at the margins of the flap on the 4th day. On the 7th day, most of the flaps survived, with small amounts of necrosis at the edges of the flaps and good hair growth on the surface of the skin (Fig. 3D). The process of change in the flaps in the Exo group was similar to that in the Hypo-Exo group, but the area of flap necrosis was larger. In addition, the flap survival rate in the Hypo-Exo group was significantly higher than that in the I/R group and Exo group (Fig. 3B). In summary, these results suggest that Hypo-Exo may be beneficial to the survival of flaps after I/R injury and that Hypo-Exo are better than Exo in improving the survival rate of skin flaps.

### Hypo-Exo reduced inflammation, ROS, apoptosis in I/R injured flaps model

**Fig. 4:**
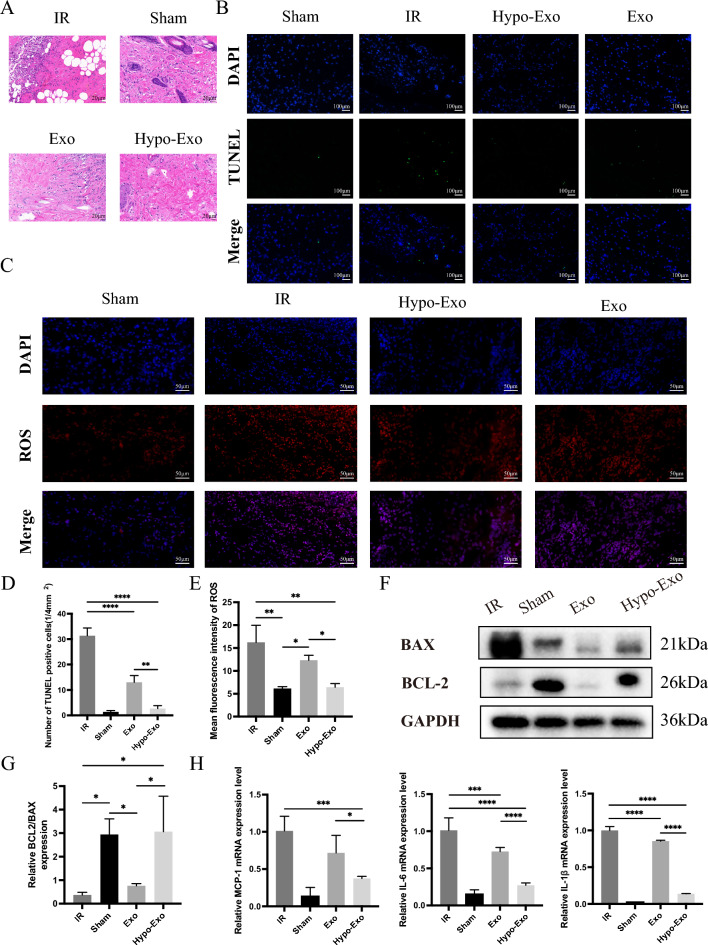
Hypo-Exo improved the pathological state of flaps after I/R injury, inhibited apoptosis, reduced the release of ROS in flap and reduced the production of inflammatory factors. **A** Representative histology images (Original magnification 40X, bar size 20 μm). **B** Detection of apoptosis by TUNEL assay (bar size 100 μm). **C** Double immunofluorescence staining of Dihydroethidium (DHE)-ROS (red) and DAPI (bule) in flap.**D** Relative quantitative data of apoptotic cells and TUNEL cells (**P < 0.01, ****P < 0.0001). **E** Statistical evaluation of ROS mean fluorescence intensities in each group (*P < 0.05, **P < 0.01). **F** Protein expression levels of Bax, Bcl-2 and GAPDH. **G** Quantitative analysis of the protein expression levels of Bax/Bcl-2 (*P < 0.05). **H** Relative MCP-1, IL-6, IL-1β mRNA expression level in skin flap was measured by qPCR in Sham, IR, Hypo-Exo and Exo groups (*P < 0.05, ***P < 0.001, ****P < 0.0001), Bars indicated means ± SD

Neutrophil accumulation, reactive oxygen species (ROS) production, and apoptosis are widely recognized as the three significant contributors to I/R injury [29]. Accordingly, this study initially examined histopathological changes in flaps. Flaps in the Sham group exhibited normal tissue structure, absence of edema, minimal neutrophil presence in the interstitium, well-structured fibers, and a continuous vessel wall. Conversely, the I/R group's subcutaneous tissue showed substantial inflammatory cell infiltration, disrupted fiber structure, and disorganized arrangement. Meanwhile, the Hypo-Exo group's flap tissues maintained overall structure integrity with only mild edema and inflammatory cell infiltration. The fiber structure remained essentially orderly, and the vessel wall was intact. The tissue structure in the Exo group closely resembled that of the Hypo-exo group but with increased inflammatory cell infiltration (Fig. 4A). The study further evaluated changes in inflammatory factor levels within the flaps. Compared to the Sham group, the I/R group exhibited significantly higher levels of MCP-1, IL-1β, and IL-6, whereas these cytokines' levels decreased in the Hypo-Exo group (Fig. 4H). As such, it was hypothesized that Hypo-Exo could reverse the upregulated production of inflammatory factors, such as MCP-1, IL-1β, and IL-6.

To delve deeper into Hypo-Exo's role in safeguarding I/R-injured flaps, ROS levels within the flaps were examined. Quantitative results illustrated that the Hypo-Exo and Exo groups had notably lower ROS levels compared to the I/R group (Fig. 4C, E).

Apoptosis levels within the flap tissue were assessed using a TUNEL assay. Virtually no TUNEL-positive cells were detected in the Sham and Hypo-Exo groups, while the I/R group had numerous TUNEL-positive cells, with a smaller number found in the Exo group (Fig. 4B, D). Echoing the TUNEL experiment's findings, Western blotting results showed that Hypo-Exo treatment upregulated anti-apoptotic protein BCL-2 expression while downregulating pro-apoptotic protein Bax expression (Fig. 4F, G). Collectively, these experiments affirm that Hypo-Exo mitigates inflammation, ROS production, and apoptosis in an I/R-injured flap model.

### Hypo-Exo protected vascular endothelial cells from I/R injured in vivo and vitro

**Fig. 5:**
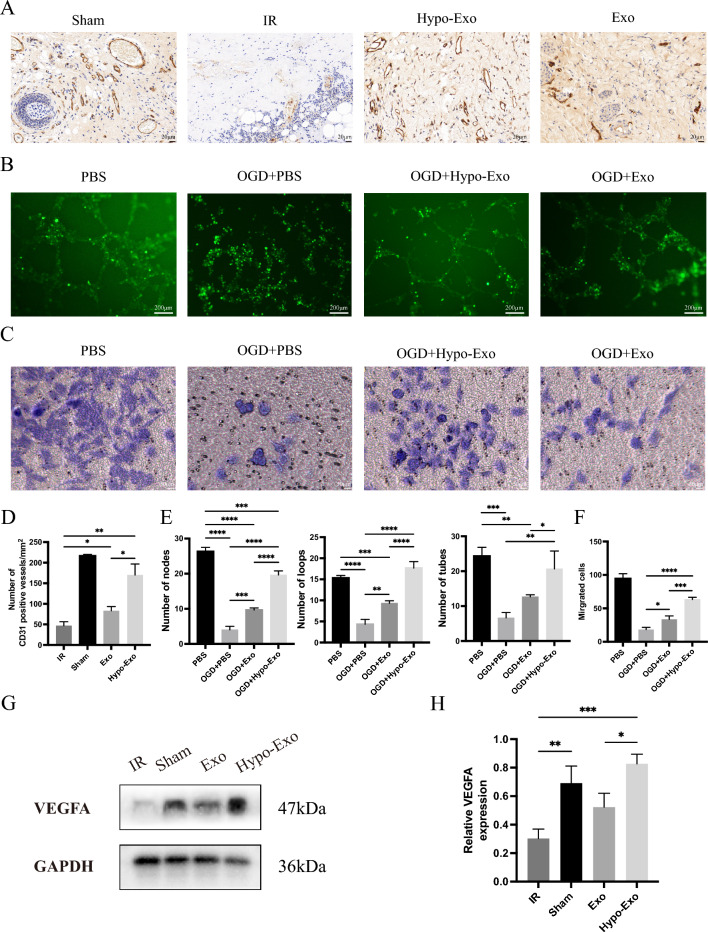
Hypo-Exo protected vascular endothelial cells from I/R damage in vivo and vitro. **A** CD31 immunohistochemical detection in each group (Original magnification 40X, bar size 20 μm). **B** Representative images showing tube formation in HUVECs treated with PBS, OGD + PBS, OGD + Hypo-Exo or OGD + Exo (bar size 200 μm). **C** Representative images showing migrated HUVECs by transwell assay treated with PBS, OGD + PBS, OGD + Hypo-Exo or OGD + Exo (bar size 50 μm). **D** The number of CD31 positive blood vessels in each group (**P < 0.01, *P < 0.05). **E** Quantitative data of tube formation using Image J (*P < 0.05, **P < 0.01, ***P < 0.001, ****P < 0.0001). **F** Quantitative data of the migrated cells (*P < 0.05, ***P < 0.001, ****P < 0.0001) **G**, **H** Western blot shows the expression of VEGF-A in flap treated with each group, and quantitative data of western blot (*P < 0.05, **P < 0.01, ***P < 0.001), Bars indicated means ± SD

I/R implicates oxidative stress, energy metabolism, inflammatory response, and cell apoptosis, all of which impact HUVECs [30]. To further elucidate the role of vascular endothelial cells in I/R injury within flaps, the quantity of CD31-positive vessels was evaluated by immunohistochemistry (IHC). Notably, the Hypo-Exo group demonstrated a substantial increase in the number of CD31-positive blood vessels compared to the I/R group. Furthermore, a significantly higher count of CD31-positive blood vessels was observed in the Hypo-Exo group in contrast to the Exo group (Fig. 5A, D). Complementary Western blotting analysis revealed a significantly enhanced expression of vascular endothelial growth factor (VEGFA) in the Hypo-Exo group relative to both the Exo group and the I/R group (Fig. 5G, H).

To ascertain if Hypo-Exo delivers therapeutic effects akin to those observed in vitro, a HUVECs-based model of oxygen glucose deprivation/reoxygenation (OGD/ROG, described as OGD in Figure) was established as previously reported [31, 32]. In this OGD/ROG model, HUVECs pretreated with Hypo-Exo and Exo formed more capillary-like structures than those in the PBS group. Quantitative results demonstrated a substantial increase in the number of tubes, loops, and nodes following stimulation with Hypo-Exo and Exo (Fig. 5B, E). Transwell assays were utilized to assess vascular endothelial cell migration capacity under OGD/ROG conditions. While the migratory capacity of HUVECs was impaired under OGD/ROG conditions, Hypo-Exo preserved HUVECs migratory capacity compared to both Exo and PBS (Fig. 5C, F). These findings suggest that Hypo-Exo outperforms Exo in protecting vascular endothelial cells from I/R damage both in vivo and in vitro.

### Exosome sequencing, miRNA differential analysis, and bioinformatics prediction

**Fig. 6:**
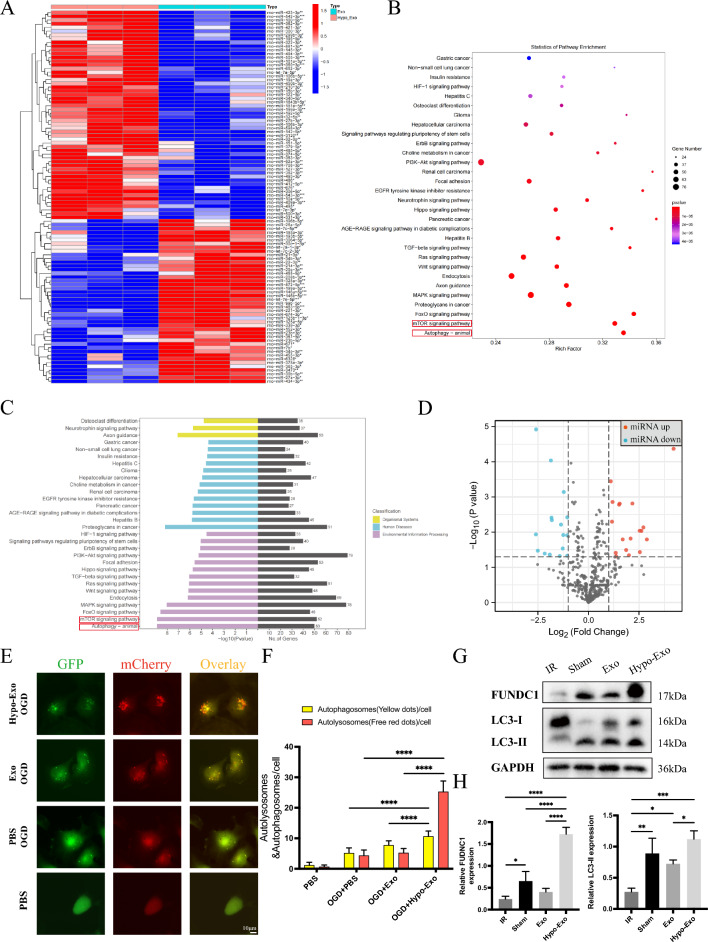
High-throughput sequencing analysis of miRNAs in Hypo-Exo and Exo. **A** Clustered heat map of differentially expressed miRNAs depicting up and down regulated miRNAs. **B**, **C** GO and KEGG enrichment of differentially expressed miRNAs. **D** Volcano plot of differentially expressed miRNAs depicting up and down regulated miRNAs. **E** Autophagosomes and autolysosomes in each group (bar size 10 μm). **F** Quantitative analysis of autophagosomes and autolysosomes in each group (****P < 0.0001). **G**, **H** Western blot shows the expression of FUNDC1, LC3-I, LC3-II in flap treated with each group, and quantitative data of western blot analysis (*P < 0.05, **P < 0.01, ***P < 0.001, ****P < 0.0001), Bars indicated means ± SD

The Exo and Hypo-Exo samples were divided into two groups and analyzed for differences in carried miRNA in terms of log2 (Fold Change) and significance level. A heat map of miRNA differential expression between the samples was constructed (Fig. 6A). Sequencing and heat map results showed that, compared to Exo, Hypo-Exo had 44 miRNAs downregulated and 56 miRNAs upregulated. To more intuitively observe the distribution of upregulated and downregulated miRNAs and to identify miRNAs with greater differences, and a volcanic plot of miRNA differences between samples was constructed (Fig. 6D). KEGG and GO biological pathway enrichment analyses were performed on miRNAs with significant differences. The results suggested that Hypo-Exo may exert potential biological effects through several biological pathways, including Autophagy-animal and the mTOR signaling pathway (Fig. 6B, C).

In order to further validate the bioinformatics prediction results and investigate whether Hypo-Exo promote flap survival by regulating autophagy, autophagy double-labeling adenovirus (mCherry-GFP-LC3) was co-cultured and transfected with HUVECs. The transfection efficiency was observed under a fluorescence microscope 24 h after transfection, showing successful transfection in over 95% of cells. Following transfection, the cells were co-cultured with Hypo-Exo, BMSC-Exo, and PBS for 24 h, and then subjected to OGD/ROG treatment. Two control groups were established with PBS: one subjected to OGD/ROG treatment and the other cultured under normal conditions. Thereafter, the number of autophagosomes and autolysosomes in each group of cells was observed under a fluorescence microscope, with red indicating autolysosomes and yellow signifying autophagosomes (Fig. 6E). The findings indicated that under normal culture conditions, the baseline autophagy level of HUVECs was very low, with an average of only 1.15 autophagosomes and 0.65 autolysosomes per cell. After 6 h of OGD/ROG treatment, the autophagy level of the cells increased, with an average of 5.15 autophagosomes and 4.35 autolysosomes observed per cell in the PBS control group. In the Hypo-Exo+OGD/ROG group, an average of 10.6 autophagosomes and 25.8 autolysosomes were produced per cell, indicating further activation of autophagy. Moreover, Hypo-Exo produced more autophagic flux apart from activating autophagy. In the Exo+OGD/ROG group, an average of 7.7 autophagosomes and 5.2 autolysosomes were observed per cell, indicating that Exo did not significantly promote autophagy compared with Hypo-Exo (Fig. 6F). These results suggest that pretreatment of HUVECs with Hypo-BMSC-Exos can activate autophagy and alleviate cell OGD/ROG damage.

Our previous study illustrated that FUNDC1 is closely related to angiogenesis and apoptosis during autophagy. Therefore, the relative expression levels of the autophagy proteins FUNDC1, LC3-I, and LC3-II were measured [33]. The results revealed that Hypo-Exo significantly upregulated the expression of the autophagy-related proteins LC3-II and FUNDC1 in I/R-exposed flaps (Fig. 6G, H). Altogether, the results showed that FUNDC1/LC3-II-related autophagy was involved in reducing ROS levels and protecting the vasculature from oxidative stress and inflammation.

### Inhibiting autophagy reversed the biological effects of Hypo-Exo in vivo and in vitro

**Fig. 7:**
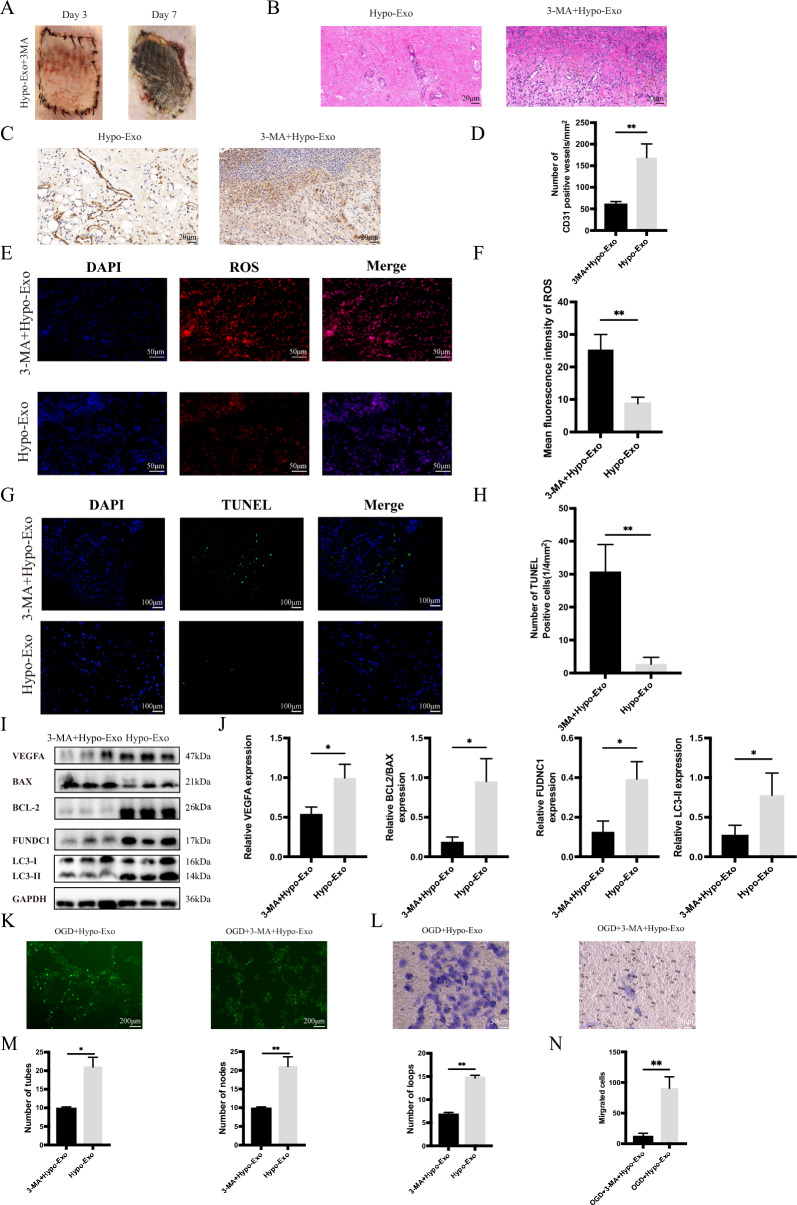
3-MA inhibit autophagy reversed the biological effects promoted by Hypo-Exo in vivo and vitro. **A** Representative skin flap appearance of each group on the7th day after operation. **B** Representative histology images of each group on the 7th day after operation **C** CD31 immunohistochemical detection in each group (Original magnification 40X, bar size 20 μm) **D** Quantitative data of CD31 positive blood vessels in each group (**P < 0.01) **E** Double immunofluorescence staining of Dihydroethidium (DHE)-ROS (red) and DAPI (bule) in each group. **F** Statistical evaluation of Mean fluorescence intensities in each group (**P < 0.01). **G** Double immunofluorescence staining of TUNEL (green) and DAPI (bule) in each group. **H** Quantitative data of apoptotic cells and TUNEL cells (**P < 0.01). **I**, **J** Western blot analysis of the expression of proteins in flap, and quantitative data of western blot (*P < 0.05). **K** Representative images showing tube formation in HUVECs treated with OGD + Hypo-Exo, OGD + Hypo-Exo + 3-MA (bar size 200 μm). **L** Representative images showing migrated HUVECs by transwell assay treated with OGD + Hypo-Exo, OGD + Hypo-Exo + 3-MA (bar size 50 μm). **M** Quantitative data of tube formation using ImageJ (*P < 0.05, ** P < 0.01). **N** Quantitative data of the migrated cells (**P < 0.01), Bars indicated means ± SD

3-MA was used to further support our hypothesis. According to previous studies, 3-MA can inhibit autophagy (including selective autophagy) through type III phosphatidylinositol (PI3K-III) [34]. Interestingly, we observed that on the 2nd day after surgery, subcutaneous bleeding spots emerged in the 3-MA+Hypo-Exo group, and on the 3rd day, the flap started to undergo necrosis and was completely necrotic at the observation endpoint (Fig. 7A). HE and IHC results showed that the 3-MA + Hypo-Exo group exhibited more inflammatory cell infiltration and necrosis, fewer CD31-positive cells and less intact tissue (Fig. 7B, C, D). In vitro experiments showed that the 3-MA+Hypo-Exo group became vulnerable to OGD/ROG damage and had lower capacities for tube formation and migration (Fig. 7K–N).

In addition, inhibition with 3-MA reversed the effect of Hypo-Exo in suppressing apoptosis and decreasing the Bcl-2/Bax protein ratio, which was also demonstrated by TUNEL assay.

(Fig. 7G–J). More apoptotic cells were found in the 3-MA+Hypo-Exo group.

We performed western blotting to compare the expression of FUNDC1 and LC3-II (Fig. 7I, J). As predicted, 3-MA+Hypo-Exo treatment significantly reduced the expression levels of FUNDC1 and LC3-II, suppressed autophagy, increased ROS levels and promoted apoptosis in the flaps (Fig. 7E, F).

### Hypo-Exo activates autophagy by suppressing mTOR via miRNA-421-3p

**Fig. 8:**
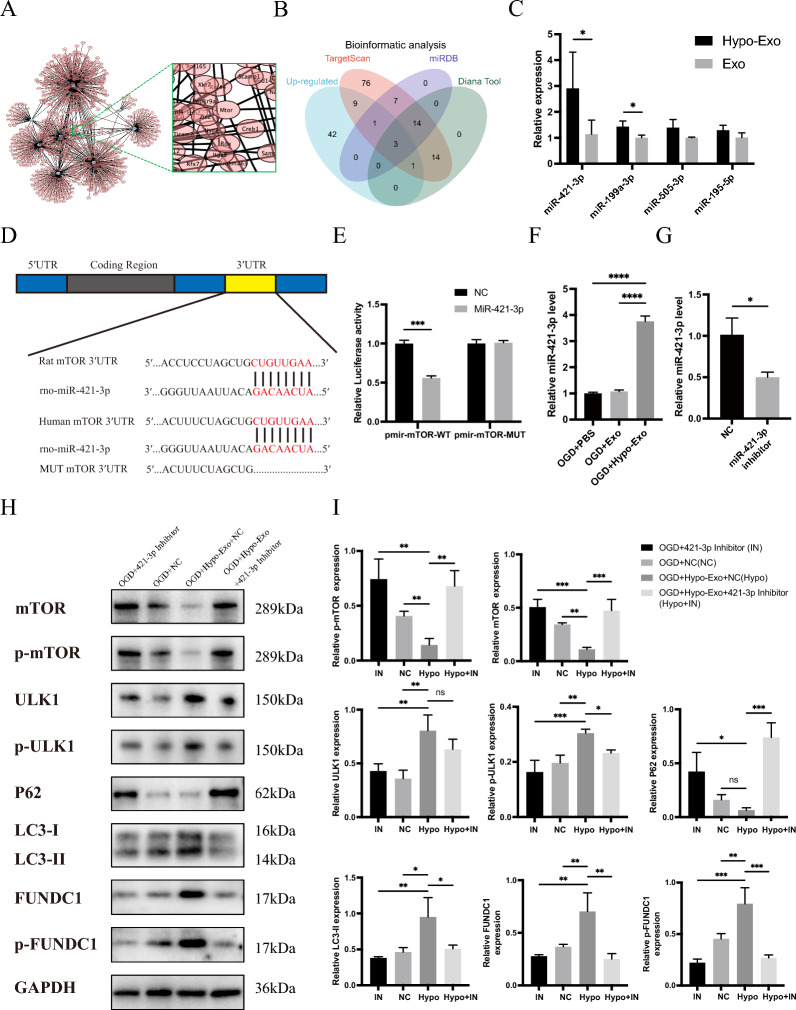
Hypo-Exo enhances autophagy by reducing mTOR protein expression by delivering miR-421-3p. **A** Network diagram for predicting miRNA target genes. **B** Bioinformatics analysis predicted miRNAs that were potentially capable of binding to mTOR. **C** qPCR analysis revealed the expression levels of miR-421-3p, miR-199a-3p, miR-505-3p, and miR-195-5p in Hypo-Exo and Exo (*P < 0.05). **D** schematic diagram illustrating the predicted binding sites of miR-421-3p in the 3'UTR of mTOR mRNA. **E** Dual-Luciferase Reporter System was used to detect luciferase activity. (***P < 0.001). **F** qPCR analysis revealed the expression level of miR-421-3p in each group (****P < 0.0001). **G** qPCR analysis revealed miR-421-3p expression levels in HUVECs after transfer with miR-421-3p inhibitors and NC (*P < 0.05). **H**, **I** Western blot shows the expression of mTOR, p-mTOR, ULK1, p-ULK1, P62, LC3-I, LC3-II, FUNDC1, p-FUNDC1 in each group, and quantitative data of western blot (*P < 0.05, **P < 0.01, ***P < 0.001), Bars indicated means ± SD

To further understand the active substances within Hypo-Exo that are responsible for activating autophagy, this study employed TargetScan database for miRNA target prediction. The results revealed multiple miRNAs within the significantly upregulated ones, that were predicted to target mTOR (Fig. 8A). In an attempt to further narrow down the scope of miRNAs, intersection was made amongst all upregulated miRNAs, employing three authoritative public databases: TargetScan, miRDB, and Diana Tool. This was then illustrated using a Venn diagram (Fig. 8B).

Incorporating sequencing expression differences and bioinformatics prediction results, this study selected miRNAs strongly associated with mTOR as predicted by multiple databases (such as miR-421-3p, miR-199a-3p, miR-505-3p, miR-195-5p) for qPCR validation. The results showed a significant difference in the content of miRNA-421-3p in Hypo-Exo compared to Exo (Fig. 8C), with miRNA-421-3p in Hypo-Exo being 2.9 times higher than in Exo, while miRNA-199a-3p also showed a 1.4 times difference. Therefore, miRNA-421-3p, which has a larger difference, was chosen for subsequent experiments. Using public databases, 3′UTR promoter sequences of mTOR in both humans and rats were identified and matched with miRNA-421-3p. The results suggested that miRNA-421-3p might bind to mTOR mRNA in humans and rats, thereby downregulating the expression of mTOR and further activating autophagy (Fig. 8D). To experimentally validate the prediction, luciferase reporter plasmids were generated. The plasmid containing the wild-type 3′-untranslated region (3′UTR) of mTOR was designated as pmir-mTOR-WT, while the plasmid with a mutated mTOR 3′UTR was labeled pmir-mTOR-MUT. Upon transfection with miR-421-3p mimics, a marked reduction in luciferase activity was observed in 293 T cells harboring pmir-mTOR-WT, in contrast to those transfected with the mutant 3′UTR plasmid (Fig. 8E). QPCR results revealed that following co-culture of Hypo-Exo and Exo with HUVECs, qPCR detected differential expression of miRNA-421-3p in the cells. Specifically, miRNA-421-3p was significantly higher in the Hypo-Exo group than in the Exo group (Fig. 8F, G). After mixing miRNA-421-3p Inhibitor, miRNA-421-3p, Inhibitor NC, and transfection reagent evenly and co-culturing with HUVECs, subsequent qPCR detection showed that miRNA-421-3p Inhibitor effectively suppressed the expression of miRNA-421-3p in the cells, with a 50% decrease in the expression of miRNA-421-3p in the miRNA-421-3p Inhibitor group compared to the miRNA-421-3p Inhibitor NC group. These experimental results suggested successful transfection and the efficacy of the miRNA inhibitor. Subsequently, the study further examined the expression of miRNA-421-3p's target mTOR. The WB results showed that after the addition of miRNA-421-3p Inhibitor, the ability of Hypo-Exo to downregulate mTOR expression was suppressed. Consequently, a decrease in mTOR phosphorylation was observed, which in turn led to an increase in the phosphorylation of ULK1 and FUNDC1 (Fig. 8H, I).

**Fig. 9:**
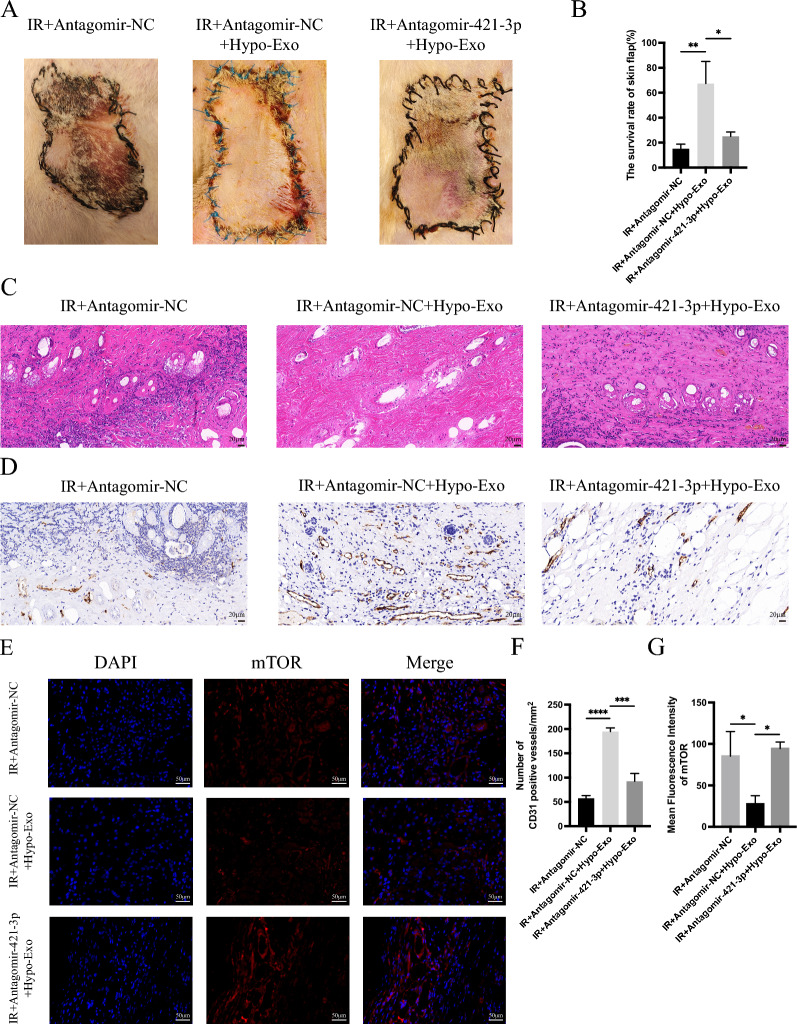
Antagomir-421-3p abolishes the therapeutic effect of Hypo-Exo. **A** Representative skin flap appearance of each group on the7th day after operation. **B** The flap survival rate of each group on the 7th day after operation (*P < 0.05, **P < 0.01). **C** Representative histology images (Original magnification 40X, bar size 20 μm). **D** CD31 immunohistochemical detection in each group (Original magnification 40X, bar size 20 μm). **E** Double immunofluorescence staining of mTOR(red) and DAPI (bule) in each group (bar size 50 μm). **F** Quantitative data of CD31 positive blood vessels in each group (***P < 0.001, ****P < 0.0001). **G** Statistical evaluation of Mean fluorescence intensities in each group (*P < 0.05). Bars indicated means ± SD

**Fig. 10:**
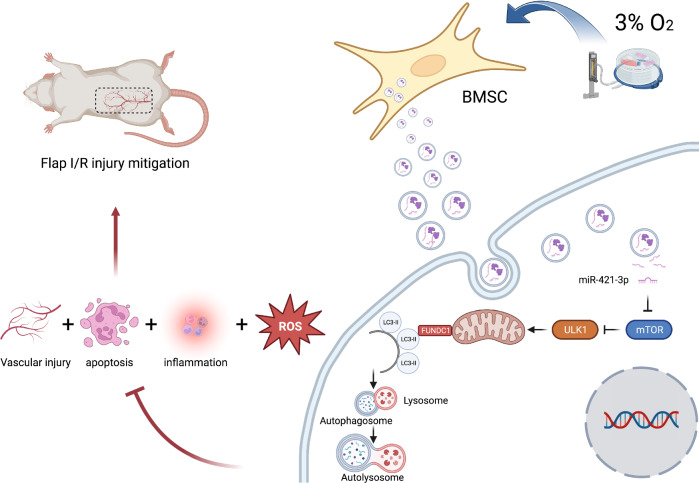
Schematic Diagram: Hypo-Exo, carrying miR-421-3p, activates autophagy by targeting mTOR and upregulating the expression of phosphorylated ULK1 and FUNDC1. This in turn mitigates the release of inflammatory factors in the skin flap, reduces ROS generation, counteracts cell apoptosis, and protects blood vessels, ultimately promoting the survival of ischemia–reperfusion flaps

In animal experiments, we further validated our hypothesis. Compared to the IR+Antagomir-NC group and the IR+Antagomir-421-3p+Hypo-Exo group, there was a significant increase in flap survival area in the IR+Antagomir-NC+Hypo-Exo group (Fig. 9A, B). HE staining and IHC results indicated reduced inflammatory cell infiltration and increased CD31-positive vessels in the IR+Antagomir-NC+Hypo-Exo group (Fig. 9C, D, F). IF results revealed that mTOR expression in the IR+Antagomir-NC+Hypo-Exo group was significantly downregulated compared to the IR+Antagomir-421-3p+Hypo-Exo and IR+Antagomir-NC groups (Fig. 9E, G). These in vivo findings corroborate our in vitro results, confirming that miRNA-421-3p inhibits the expression of mTOR (Fig. 10).

## Discussion

Free flaps are commonly used in the treatment of tissue defects in microsurgery and reconstructive surgery. However, the rate of partial flap necrosis has been reported to reach up to 7–20% [5, 35]. Skin flap necrosis after flap transplantation is primarily caused by ischaemia–reperfusion (I/R) injury, which is thought to be associated with the generation of reactive oxygen species, significant neutrophil accumulation, vascular endothelial cells injury and apoptosis [29]. Exosomes derived from mesenchymal stem cells have been considered to be effective tools to treat a variety of refractory clinical diseases ever since the development of regenerative medicine [36–38]. However, the biological effects of exosomes are determined by the state of the source cells, and hypoxia pretreatment has been reported to increase the therapeutic effects of MSC. According to previous reports, oxygen concentrations in the bone marrow cavity can be as low as 3% in mammals [39], which is very different from the oxygen concentrations that are present when BMSCs are cultured in vitro (O^2^ levels of 21%). Thus, we chose to expose BMSCs to hypoxia and hoped that exosomes derived from hypoxic BMSCs could assist the cells in I/R flap tissues to further promote flap survival. Based on these considerations, we designed a stable ischaemia–reperfusion flap model in rats and hypothesized that (1) exosomes derived from hypoxia-pretreated BMSCs would exert better biological effects than those derived from cells cultured under normal oxygen concentrations; (2) Hypo-Exo would promote flap survival by reducing inflammation, ROS production and apoptosis; (3) Hypo-Exo would protect vascular endothelial cells from I/R injury.

In this work, instead of using microvascular clamps, we performed microsurgical anastomoses to induce I/R injury. The clamp model is prone to thrombosis, causes vasospasm, and is less translatable to clinical scenarios in free flap surgery [40]. Microsurgical anastomoses are time-consuming and require fine microsurgical skills, and we perfused five millilitres of heparinized normal saline (5000 U/L) under minimal constant during the surgery, which effectively prevents thrombosis. After performing end-to-end anastomoses on the animals in the I/R group, indocyanine green angiography was utilized to intraoperatively assess blood flow patency and to verify blood flow 1 week later. Blood flow was also measured in the Sham group on the day of the surgery, and at the end of the study, no animal's blood flow was impaired by the anatomical dissections, vascular ligatures, or anastomoses. Furthermore, we did not notice any pain or limping as a result.

To understand whether exosomes derived from hypoxia-pretreated BMSCs exert better biological effects, we studied the morphology of Hypo-Exo and Exo by TEM. Although there was not much difference between Hypo-Exo and Exo, we observed that hypoxia could promote BMSC exosome release, and there was a higher total protein concentration in the Hypo-Exo samples. The experiment on the internalization of exosomes revealed that Hypo-Exo were more easily taken up by HUVECs, which is consistent with previous studies [17, 41]. According to live imaging, we found that 24 h after the local injection of equal volumes and concentrations of DiR-labelled Hypo-Exo and Exo, the Hypo-Exo group had higher fluorescence intensity in the flap region, which may suggest Hypo-Exo are more easily taken up in flaps, possible because hypoxia changes the abundance of proteins on the exosome surface.

Previous studies have shown that different types of MSCs can reduce ischaemia–reperfusion injury under hypoxic conditions [17, 42, 43]. We further explored whether Hypo-Exo derived from BMSCs can reduce free flap injury and the specific mechanisms involved. We found that Hypo-Exo successfully and significantly promoted flap survival after 7 days, and we attempted to understand how they reduced I/R injury. Inflammation, reactive oxygen species production, and apoptosis are thought to be the three factors that significantly contribute to I/R injury [29]. In our study, we first assessed the differences between the groups in terms of inflammation. MCP-1, which is primarily released by monocytes, macrophages, and dendritic cells, functions as a monocyte chemotactic protein. When an inflammatory response occurs, it stimulates leukocyte adhesion molecule-1 expression, leukocyte chemotaxis, and IL-1β and IL-6 cytokine production [44]. We confirmed that the relative expression levels of MCP-1, IL-6, and IL-1β were increased in I/R-exposed flaps; however, Hypo-Exo and Exo reduced inflammatory factor production. Moreover, HE showed less neutrophil accumulation in the Hypo-Exo group and maintained a better tissue structure.

We also examined ROS levels in the flap tissues after 7 days. Reactive oxygen species generation occurs within the first few minutes of reperfusion. Activation of the electron transport chain during the restoration of blood flow increases ROS production. Intravascular endothelial cell metabolism, which is induced by xanthine oxidase, generates ROS. Hours after reperfusion, neutrophils accumulate at the necrotic site and can also generate ROS [45]. We observed that Hypo-Exo reduced the ROS levels in the I/R-exposed flaps, and therefore, we carried out a follow-up experiment to investigate the specific mechanism involved. In general, reduced ROS levels are further evidence that Hypo-Exo can attenuate ischaemia‒reperfusion injury. Finally, we evaluated indicators that are related to apoptosis. Apoptosis is a mode of cell death that occurs under physiological and pathological conditions. This concept was first proposed by the American pathologist Kerr et al. [46] in 1972, and inhibition of apoptosis can promote flap survival. Two major proteins that are crucial for controlling apoptosis are Bcl-2 and Bax. WB revealed that Hypo-Exo significantly increased the Bcl-2/Bax ratio and that the Hypo-Exo group had fewer TUNEL-positive cells. Apoptosis was inhibited in I/R-exposed flaps, further confirming that Hypo-Exo protects against I/R damage.

Hypoxia-preconditioned BMSCs have been reported to enhance angiogenesis [41, 47, 48]; however, few studies have demonstrated whether Hypo-Exo can protect vascular endothelial cells from I/R injury. IHC revealed more CD31-positive vessels in the Hypo-Exo group in vivo. To better understand how Hypo-Exo help HUVECs tolerate I/R injury in vitro, we designed a 6-h 3% O^2^ OGD/ROG model that accurately simulates ischaemia‒reperfusion injury in flaps. Transwell and tube formation assay results showed that both Hypo-Exo and Exo could protect HUVECs from OGD/ROG damage, and Hypo-Exo were even more effective. However, some studies have shown that 15–30 min of hypoxia treatment is beneficial to vascular endothelial cells [3, 49]. We believe that sufficient glucose and short-term exposure to hypoxia may be beneficial, but glucose deprivation and long-term exposure to hypoxia lead to apoptosis. All of these experiments demonstrated similar results, suggesting that Hypo-Exo reduced neutrophil accumulation, prevented vascular endothelial cell and other cell apoptosis and ameliorated the generation of reactive oxygen species. Thus, we confirmed our hypothesis that Hypo-Exo protected skin flaps by reducing inflammation, ROS production, and apoptosis and protecting HUVECs.

MiRNAs are a class of endogenous noncoding RNAs that are approximately 19–22 nucleotides long; they can bind to complementary sites in the 3′-untranslated regions (3′UTRs) of their target miRNAs and negatively regulate protein-coding gene expression [50]. Studies have demonstrated that miR-126 aids in the recovery from bone fractures. [17], miR-612 can enhance angiogenesis [47], and miR-486 can promote angiogenesis after myocardial infarction [48]. So, we sequenced Hypo-Exo and Exo at a high throughput. According to data from high-throughput sequencing, 56 microRNAs (miRNAs) were elevated while 44 miRNAs were downregulated. A previous study reported that autophagy can protect BMSCs from apoptosis under hypoxic and serum-deficient conditions [51]. We hypothesize that exosomes derived from hypoxia-pretreated BMSCs may carry autophagy-related miRNAs to assist HUVECs and other cells in adapting to hypoxic environments and to prevent apoptosis. Bioinformatics analysis further confirmed our hypothesis, and the results of GO and KEGG analyses indicated that exosomes released by BMSCs under hypoxic conditions exerted their regulatory effects partially by activating autophagy pathways.

Autophagy, which is largely conserved across evolutionary time, can prevent or postpone apoptosis in response to various stresses such hunger, DNA damage, and physiological pressure. Increasing numbers of studies have indicated that autophagy protects cells from harmful stimuli, allowing them to die only after sustained stimulation [52–54]. ROS are direct causes of oxidative stress, and 90% of them come from the inner mitochondrial membrane's respiratory chain. Numerous investigations have demonstrated that the primary inducers of autophagy during oxidative stress are ROS that are generated in mitochondria. By modulating several signalling pathways during the production of autophagosomes, ROS can trigger autophagy [54]. Thus, according to the immunofluorescence results, we reasonably speculate that the differences in the ROS levels in the different groups might be related to the level of autophagic activity. According to the 2021 Fourth Edition Guidelines for Autophagy [55], a dual-labeled autophagy adenovirus serves as an excellent tool for detecting autophagy. It allows for the assessment of autophagy activation level as well as the evaluation of the extent of autophagic flux. Therefore, it was initially confirmed in vitro that Hypo-Exo can activate autophagy in HUVECs, thereby alleviating OGD/ROG damage. At the same time, we selected several autophagy-related antibodies that our laboratory had already owned for validation. Among these autophagy-related proteins, we found that the changes in FUNDC1 and LC3-II were particularly pronounced. In the Hypo-Exo group, we observed upregulation of LC3-II and FUNDC1, which are widely accepted markers of autophagy [56, 57]. In addition, FUNDC1 is a novel mitochondrial protein that promotes angiogenesis, antioxidative stress processes [58, 59] and anti-apoptosis processes. To further understand whether Hypo-Exo-mediated autophagy is important for reducing ROS production, inflammation and apoptosis and protecting HUVECs, we co-treated Hypo-Exo with the autophagy-specific inhibitor 3-MA. As we mentioned before, 3-MA can inhibit autophagy (including selective autophagy) through type III phosphatidylinositol (PI3K-III) [34]. The in vivo and in vitro results suggest 3-MA completely reversed the biological effects of Hypo-Exo, which reinforces the fact that Hypo-Exo-mediated promotion of (I/R)-injured flap survival involves the activation of autophagy pathways.

To further understand the biologically active components within Hypo-Exo responsible for autophagy activation, a comprehensive analysis of differentially expressed miRNA in Hypo-Exo and Exo was carried out. Bioinformatic analytic techniques were employed to construct a miRNA target gene network. The findings from this network indicated multiple targeting instances on mTOR. qPCR results indicated a significant enrichment of miR-421-3p in Hypo-Exo. We next validate whether miR-421-3p could inhibit mTOR expression through both in vitro and in vivo experiments. Luciferase reporter assay and Western blot assays confirmed that miR-421-3p inhibits mTOR translation by binding to the 3′UTR of mTOR mRNA. Furthermore, in animal experiments, the administration of antagomir-421-3p reversed the ability of Hypo-Exo to downregulate mTOR expression. Collectively, these findings substantiate that miR-421-3p carried by Hypo-Exo can specifically target and modulate mTOR expression, corroborating prior study [60]. mTOR primarily exerts regulatory influence on autophagy via the action of mTORC1, with the phosphorylation of mTOR itself being indispensable for mTORC1 activation [61]. Given sufficient growth factors, nutrients, energy, and oxygen, mTORC1 becomes activated, instigating cell growth and division. Concurrently, mTORC1 phosphorylates and inactivates the ULK1 (Unc-51-like kinase 1) complex, effectively impeding the onset of autophagy. FUNDC1, a novel mitochondrial membrane protein, is recognized for its close association with mitochondrial quality control and the autophagy process. Mitophagy, a specialized form of autophagy, entails the removal of damaged or surplus mitochondria via the autophagic pathway [62]. As a mitochondrial membrane protein, FUNDC1 plays a pivotal role in mitophagy regulation [63]. Previous research has shown that FUNDC1 can engage with ULK1 and initiate mitophagy under hypoxic conditions [64]. ULK1 promotes the interaction between FUNDC1 and LC3 via phosphorylation, thereby augmenting mitophagy [21]. When mTORC1 activity is elevated, autophagy is suppressed. Conversely, under conditions of inadequate nutrients and energy, a decrease in mTORC1 activity permits the dissociation and activation of the ULK1 complex. The active ULK1 complex can facilitate autophagosome formation and regulate FUNDC1 activity through phosphorylation, hence promoting autophagy. In such conditions, autophagy can aid cell survival under stress.

While the current study provides valuable insights, several limitations should be noted. Given the precision and complexity required in microsurgical procedures, variables in the surgical process were controlled by having all ischaemia–reperfusion flap models in rats performed by a single microsurgeon, which inherently restricted the capacity for larger sample sizes. Moreover, the mTOR-regulated autophagy signaling pathway might not be the sole conduit through which Hypo-Exo promote flap survival. In addition to miR-421-3p, other miRNAs such as miR-199a-3p could potentially participate in autophagy regulation. This study did not venture into more extensive investigations of other microRNAs concerning autophagy and other pathways.

Although both HUVECs and rats were utilized for validation in this study, it should be pointed out that apart from vascular endothelial cells, other cell types in the flap may also contribute to some extent. Single-cell sequencing technology could be a potential research tool to help further comprehend the roles played by different cell types in ischaemia–reperfusion. Simultaneously, it is recognized that results derived from cell experiments, animal studies, and clinical applications may harbor uncertainties. Before clinical application, the efficacy and safety of Hypo-BMSC-exos in different ischaemia–reperfusion animal models need further verification.

Additionally, issues related to the standardization of exosome purification methods and the ethics of using human-derived cells still need to be addressed. Finally, further research is warranted to understand the dosage, route of administration, and potential impacts of exosomes on the human body in clinical applications.

## Conclusions

The present study demonstrates that Hypo-Exo, enriched with miRNA-421-3p, could inhibit mTOR expression, upregulate the phosphorylation of ULK1, thereby increasing the phosphorylation of FUNDC1, and further activate autophagy. This autophagy activation can reduce the release of inflammatory factors in the skin flap, decrease ROS generation, counteract cell apoptosis, and protect blood vessels, ultimately promoting skin flap survival.

## Methods

### Treatment for hypoxia and cell culture

HUVECs were purchased from the Chinese Academy of Sciences' Cell Bank (Shanghai, China). The cells were cultured in 1640 medium (BasalMedia, China) supplemented with 10% FBS (Gibco, USA) and 1% penicillin–streptomycin solution (Biosharp, China) at 37 ℃ in a 5% CO^2^ humidified atmosphere. Rat bone marrow mesenchymal stem cells (BMSCs) were purchased from HonorGene (Changsha China). BMSCs were cultivated in low-glucose Dulbecco’s modified Eagle's medium (DMEM; BasalMedia, China) with 10% fetal bovine serum (FBS; Gibco, USA) and 1% penicillin–streptomycin solution (Biosharp, China) supplements at 37 ℃ in a 5% CO^2^ humidified environment. For hypoxic preconditioning, BMSCs were transferred into a hypoxic workstation (DWS, UK) and cultured in 3% O^2^, 92% N^2^ and 5% CO^2^ for 24 h. Conditioned media was obtained for exosome isolation under both normoxic and hypoxic conditions.

### Exosome isolation and identification

BMSCs were grown under either normoxic or hypoxic conditions for an additional 24 h after the medium was replaced with culture medium containing exosome-depleted FBS when they had reached 80% confluence. Then, the culture medium was collected and centrifuged at 4000 × *g* for 30 min. After centrifugation, the cell supernatants were separated from the whole cells and cellular debris using a 0.22 μm sterile filter (Steritop TM Millipore, USA). The filtered supernatants were then added to the top compartment of an Amicon Ultra15 Centrifuge Filter Unit (Millipore, USA), and they were centrifuged at 4000 × *g* for another 30 min until the upper compartment's volume was almost 300 µl. The ultrafiltered supernatants were then refiltered to another 300 µl after being washed twice with PBS. The filtered supernatants transferred to a sterile vessel, and the appropriate volume of ExoQuick-TC (SBI, USA) was added to the filtered supernatants. The samples were mixed well by inverting or flicking the tube, and then, they were incubated in a refrigerator overnight. The ExoQuick-TC/supernatant mixtures were centrifuged at 1500 × *g* for 30 min before the supernatants were aspirated. The residual ExoQuick-TC solution was spun down by centrifugation at 1500 × *g* for 5 min. After this, all traces of fluid were removed by aspiration, and the exosome pellets were resuspended in 300 µl using sterile 1X PBS. Exosomes were either used immediately for further experiments or promptly stored at – 80 ℃. Nanoparticle tracking analysis (Zetaview-PMX120-Z, Particle Metrix, Germany) and transmission electron microscopy (Hitachi, Japan) were used to evaluate the shape and size distribution of the exosomes. A BCA Protein Assay Kit (Multi Sciences, China) was used to measure the protein concentrations. With the use of western blotting, the expression of exosome surface markers was detected.

### Internalization of exosomes

Hypo-Exo and Exo were labelled with Dio dye (Abbkine Scientific, China) to show exosome internalization by HUVECs. HUVECs were cocultured with Dio-Exo and Dio-Hypo-Exo for 6 h, after which the HUVECs were stained with DAPI (Beyotime Biotechnology, China). To observe the signals in cells, a fluores-cence microscope (Leica, Germany) was employed.

### Oxygen–glucose deprivation and reoxygenation and reglucose (OGD/ROG)

HUVECs were cultured for 6 h at 37 ℃ in media deprived of serum and glucose, 3% O^2^, 92% N^2^ and 5% CO^2^ in a hypoxic workstation (DWS, UK) to induce OGD/ROG. Previously to OGD, HUVECs were cultured in a medium containing Hypo-Exo, Exo (100 μg/mL), and PBS. The cells were then re-incubated for 24 h under standard cell culture conditions (reoxygenation and reglucose, ROG) for subsequent experiments.

### Transwell assay

To a 24-well Transwell plate (Corning, NY, USA; pore size: 8 m), 2 × 10^4^ HUVECs or OGD/ROG-HUVECs were seeded in the top chamber, and 500 μL/well medium was added to the bottom chamber after different treatments (100 µg/mL Hypo-Exo, 100 µg/mL Exo, 5 mM 3-MA or PBS). After coincubation for 36 h, cells were wiped off the top of the filter membrane using a cotton swab. Crystal violet (0.5%) was used to stain the cells on the bottom surface of the filter membrane for one minute. By using an optical microscope (Leica, Germany) to examine the stained HUVECs, migratory activity was evaluated by calculating the number of crystal violet-positive cells with ImageJ (National Institutes of Health, USA).

### Tube formation assay

HUVECs or OGD/ROG-HUVECs (5 × 10^4^) were added to 48-well culture plates that were coated with Matrigel (130 μL/well, BD Biosciences, USA) in basic medium with different treatment(100 μg/mL Hypo-Exo, 100 μg/mL Exo, 5 mM 3-MA or PBS). Tube formation was observed in at least three fields after 8 h of incubation using a fluorescence microscope (Leica, German). The numbers of nodes, loops, and tubes were measured using ImageJ (National Institutes of Health, USA).

### Animal experiments

Thirty 12-week-old male Sprague‒Dawley rats (Beijing Huafukang Biotechnology Co., Ltd. China) weighing 350–400 g were used in the experiment. A temperature-controlled environment with a 12/12-h light–dark cycle and unlimited access to food and water were provided for the animals. All of the experiments used in this study received approval from the Xiangya Hospital Ethics Committee at Central South University and adhered to all guidelines established by the Chinese Animal Care and Use Committee.

### Flap model and experimental design

An intraperitoneal dose of the anesthetic sodium pentobarbital (30 mg/kg) was given to each rat. The animals were given a deep anesthetic, had their stomachs and groins shaved, and were then laid. The free inguinal flap model was used in the experiment. After dissection was completed the Sham group, the rats were resutured without being subjected to ischaemia. In the ischaemia–reperfusion group and the experimental group, according to the method described Alberto Ballestín and Kazuya Odake et al. [8, 40, 65], a 3 × 6 cm^2^ free inguinal flap was generate at the base of the right upper abdominal artery and vein, the femoral artery was severed, and the femoral vein was induced to induce ischaemia (Fig. 2A, B, C). Additionally, the flap was perfused with heparin saline. Following 6 h of ischaemia, the femoral artery was sewn using 10–0 nylon suture, and the femoral vein was utilized to restore the flap's blood supply. The flap was sutured in place once the blood flow had been restored. All the groups received subcutaneous injection of enrofloxacin (7.5 mg/kg/day for 3 days) to prevent infection and subcutaneous injection of meloxicam (1 mg/kg/day for 3 days) to reduce pain. In both the Hypo-Exo and Exo groups, rats received a daily injection of 200 µg of exosomes in the flap region for seven consecutive days, Intradermal injections were administered at four distinct locations on the skin flap using insulin injection needles, in Hypo-exo+3-MA group, 3-MA (10 mg/kg) intraperitoneally for 7 consecutive days. The flap was divided into 6 areas; area 1 was used for histological analysis, immunohistochemical analysis and immunofluorescence analysis, and area 4 was used for qPCR and western blotting analysis.

### In vivo* imaging of DIR-labeled exosomes in flap*

Briefly, DIR-labeled Hypo-Exo and Exo were locally injected at four points along the edges of the skin flap on the day of the surgery. Each injection contained 2 µg/µl of fluorescent exosomes in a total volume of 100 µl. The distribution of the two types of exosomes in the skin flaps of the two groups of rats was observed by in vivo imaging 24 h later. The Lumina IVIS Spectrum Imaging System (PerkinElmer, USA) was used for in vivo imaging, capturing the fluorescent signals through filters of 750 nm and 782 nm. The fluorescent images were analyzed using Living Image software (PerkinElmer, USA).

### Flap assessment

Indocyanine green was injected into the dorsal vein of the penis on the day after operation and on the 7th day. The flap's blood flow and tissue perfusion were evaluated using a laser imaging system (Harbin Haihong Foundation Technological Development Co., Ltd. China). Using Image J software for image analysis. The viable flap area was calculated was calculated as a percentage using the formula: (cm^2^ of viable area/cm^2^ of total valve area) 100%.

### Histological analysis

On the 7th post-operative day, 4% paraformaldehyde was used to fix the flap sample samples. The samples were dehydrated and paraffin-embedded after 24 h. After deparaffinization with xylene and ethanol, samples of flap tissue were stained with hematoxylin and eosin. The manufacturer's recommended method for HE staining was followed. Afterwards, images were taken under a microscope for examination.

### Western blotting analysis

The proteins were extracted with RIPA buffer, separated on polyacrylamide gels using SDS, and then electrotransferred onto membranes made of polyvinylidene fluoride (PVDF). After incubating the membranes for 20 min in NcmBlot blocking buffer (NCM Biotech, China), they were probed overnight at 4 ℃ with primary antibodies specific for the proteins listed below: CD63, CD9, CALENXIN (Santa Cruz Biotechnology, Santa Cruz, USA), VEGFA, FUNDC1, p62, LC3, BCL-2, BAX, and GAPDH (Abcam, USA), mTOR, p-mTOR, ULK1, p-ULK1 (Cell Signaling Technology, USA), p-FUNDC1(Affinity Biosciences, CHINA). Antibody reactivity was detected using an ECL kit (Biosharp, China) and visualized using the ChemiDocTM XRS+system (BIO-RAD, USA).

### Measurement of ROS levels in flap tissues

For staining, frozen sections were warmed, outlined with a histochemical pen, and then incubated for 30 min at 37 ℃ in the dark with DHE diluted in PBS (generally PBS: DHE = 200:1). Slides were submerged in PBS (pH 7.4), rinsed three times for 5 mins each, and then stained to show the nuclei. The slides were next stained for 10 min at room temperature and in complete darkness using DAPI staining solution. The slides were washed three times in PBS (pH 7.4) for 5 mins each to seal the samples. The sections were then shaken dry and sealed with anti-fluorescence quenching sealer. The slices were observed and pictures were taken using a Nikon inverted fluorescent microscope for microscopic investigation. ImageJ measured the average ROS florescence intensity.

### Immunofluorescence analysis

To rehydrate tissue sections, we first deparaffinized them in xylene and then subjected them to a series of ethanol washes of varying strengths. Antigen retrieval (20 min at 95 ℃, 10.2 mM sodium citrate buffer) was performed on the washed sections. The sections were blocked with 3% goat serum for 30 min at room temperature after being rinsed in PBS (pH 7.4) three times for 5 mins each. The slices were treated with TUNEL reagent or mTOR overnight at 4 ℃ (As for mTOR the primary antibody was diluted to the specified concentration using antibody dilution buffer and incubated overnight at 4 ℃, the secondary antibody was prepared in a 1:100 dilution with phosphate-buffered saline with Tween-20 (PBST) and incubated for 1 h at 37 ℃). After being washed three times for 5 mins at room temperature, the sections were incubated with DAPI staining solution for a further 10 mins. Fluorescence microscopy was used to examine the sections (Nikon Eclipse, Japan).

### Immunohistochemical analysis

First, the tissue blocks are deparaffinized in xylene, followed by rehydration. Then, a 3% hydrogen peroxide solution is applied to the sections. Heat-induced antigen retrieval is performed for 10 min at 95 degrees Celsius (pH 6.0). The samples are then cooled to room temperature. Subsequently, the sections are blocked with 3% BSA for 30 min and incubated overnight with the primary antibody anti-CD31 at 4 ℃. After incubation with a secondary antibody conjugated with horseradish peroxidase, staining is performed using a DAB detection kit and counterstained with hematoxylin. Using the Image-Pro Plus program, CD31 expression absorption values were computed, and immunohistochemistry (IHC) statistical analyses were then performed.

### Small RNA sequencing

MagZol (Magen) was used to extract total RNA from exosomes according to the manufacturer's instructions. Quantity and quality of RNA were evaluated with the use of the K5500 and Agilent 2200 TapeStation (Agilent Technologies, USA). In a nutshell, RNAs were first ligated to a 3′ RNA adapter, then to a 5′ RNA adapter. The RNAs that had been ligated to adapters were then subjected to RT-PCR amplification at a low cycle. Size selection of the PCR products was next performed by polyacrylamide gel electrophoresis (PAGE) as described in the NEBNext^®^ Multiplex Small RNA Library Prep Set for Illumina^®^ protocol. (Il-lumina, USA). Agilent 2200 TapeStation was used to analyze the cleaned-up library items. At Ribobio Co., Ltd., we used a HiSeq 2500 (Illumina, USA) to sequence the libraries using a single-end run of 50 bp. (Ribobio, China).

### Bioinformatics analysis

After processing the raw data with FASTQC, which eliminated adapter-containing reads, poly "N" reads, low quality reads, and reads shorter than 17 nt, clean readings were produced. BWA was used to map clean reads to the reference genome in order to produce mapping reads.Mature miRNAs were identified using miRBase21 (www.miRBase.org), and new miRNAs were predicted using miRDeep2. MiRNA expression was calculated using reads per million (RPM) measurements [RPM = (number of reads mapping to miRNA/number of reads in Clean data) 10^6^].RPM, which equals (number of reads mapping to miRNA/number of reads in Clean data)10^6^, was used to standardize the expression levels. The edgeR algorithm determined the differential expression between two sets of data based on the following criteria: |log2(Fold Change)|1 and P value 0.05.Many approaches, including TargetScan, miRDB, miRTarBase, and miRWalk, were used to predict the target genes for a subset of miRNAs. KOBAS was used to do further pathway analysis utilizing GO and KEGG (Kyoto Encyclopedia of Genes and Genomes). Differentially expressed miRNAs and their respective target genes were incorporated into Cytoscape to create a miRNA network. Additionally, miRNAs predicted to target mTOR, sourced from different databases, were depicted in a Venn diagram.

### Assessment of autophagic flux

HUVECs were seeded in a 24-well plate inside a biosafety cabinet. Upon reaching a confluence of 50%, the cells were transfected with mCherry-GFP-LC3 adenovirus. The multiplicity of infection (MOI), representing the number of infectious virus units per cell, was maintained around 200. Following a 6-h transfection period, the number of transfected cells was observed under a fluorescence microscope. Upon successful transfection, the medium was replaced. Subsequently, Hypo-Exo, Exo and PBS were added to each group and then subjected to Oxygen Glucose Deprivation/Reoxygenation (OGD/ROG) treatment or left untreated. Post intervention, autophagic flux was observed and imaged under high magnification via a fluorescence microscope.

### Luciferase reporter assay

Luciferase Reporter constructs containing either the wild-type 3′-untranslated region (3′UTR) of mTOR (designated as pmir-mTOR-WT) or its mutant form (pmir-mTOR-MUT) were generated. 293 T cells were transfected with these constructs alongside miR-421-3p mimics using lipofectamine TM2000 (Invitrogen, USA). After a 48-h incubation period, luciferase activities were quantified using the Dual-Glo Luciferase Assay System (Promega, USA), following the manufacturer's guidelines.

### MiRNA inhibitor transfection

The miRNA inhibitor, inhibitor NC, and transfection reagent, riboFECT^™^ CP Reagent, were procured from RiboBio (RiboBio, China). Following the manufacturer's instructions, the recommended doses of riboFECT^™^ CP Buffer miRNA inhibit and NC were mixed. The transfection reagent riboFECT^™^ CP Reagent was then added to the mixture, gently vortexed to prepare the transfection complex. In a biosafety cabinet, HUVECs were seeded in a 12-well plate and once the cell confluence reached about 50%, the medium was replaced with antibiotic-free culture medium. The prepared transfection complex was then added according to the manufacturer's recommended dosage, and cells were incubated for 24 h before subsequent treatment. The success of transfection was evaluated using qPCR and Western blot. The antagomir-421-3p, antagomir-NC were procured from RiboBio (RiboBio, China), In the animal experiments, antagomir-421-3p at a dose of 10 nmol and Hypo-Exo was administered via subcutaneous injection on the first and 4th days post-surgery. The control group received subcutaneous injections of antagomir-NC at a dose of 10 nmol."

### Q-PCR

According to the manufacturer's instructions, the SteadyPure RNA Extraction Kit (Accurate Biotechnology (Hunan) Co., Ltd. China) was used to isolate total RNA from rat skin flaps and HUVEC cells. miRNAs were isolated from Exo and Hypo-Exo using the Exosomal RNA Purification Kit (Genecopoeia, Guangzhou, China). For real-time PCR analysis of mRNA, cDNA was synthesized using Evo M-MLV Reverse Tran-scription Kit (Accurate Biotechnology (Hunan) Co., Ltd. China), followed by Premix Pro Taq HS qPCR Kit (Accurate Biotechnology (Hunan) Co., Ltd. China) and gene-specific primers for real-time PCR. For real-time PCR analysis of mature miRNA, miRNA 1st strand cDNA synthesis kit (Accurate Biotechnology (Hunan) Co., Ltd. China) was used for miRNA cDNA synthesis, followed by Premix Pro Taq HS qPCR Kit (Accurate Biotechnology (Hunan) Co., Ltd. China) and gene-specific primers for real-time PCR. According to the 2^-ΔΔCT^ comparison method, the relative mRNA expression level was normalized to the expression level of GAPDH, and the relative expression level of miRNA was normalized to the expression level of U6. Primers for mRNA and miRNA analysis were purchased from Accurate Biotechnology (Changsha, China). miRNA qPCR 3′primer provided with miRNA 1st strand cDNA synthesis kit(Accurate Biotechnology (Hunan) Co., Ltd. China).Table 1 contains a list of the primers used in qPCR.

**Table 1:** primers used for Q-PCR

Primer name	Sequence (5′–3′)
MCP-1 forward	CTATGCAGGTCTCTGTCACGC
MCP-1 backward	CAGCCGACTCATTGGGATCA
IL-1β forward	TTGAGTCTGCACAGTTCCCC
IL-1β backward	GTCCTGGGGAAGGCATTAGG
IL-6 forward	CACTTCACAAGTCGGAGGCT
IL-6 backward	AGCACACTAGGTTTGCCGAG
GAPDH forward	GCATCTTCTTGTGCAGTGCC
GAPDH backward	GATGGTGATGGGTTTCCCGT
miR-421-3p forward	GGATCAACAGACATTAATTGGG
miR-199a-3p forward	ACAGTAGTCTGCACATTGGTTA
miR-505-3p forward	GTCAACACTTGCTGGTTTCC
miR-195-5p forward	TAGCAGCACAGAAATATTGGC
U6 forward	GGAACGATACAGAGAAGATTAGC
U6 backward	TGGAACGCTTCACGAATTTGCG

### Statistics

Statistical analysis was performed using SPSS software (ver. 25.0; SPSS, Chicago, IL). All the data are expressed as the mean and standard deviation. Comparisons between two groups were made using the independent samples t test, whereas comparisons between three or more independent groups were made using one-way analysis of variance. P values under 0.05 were regarded as statistically significant in all analyses.

## Data Availability

All relevant data and materials are available from the authors upon reasonable request.

## Abbreviations

I/R: Ischaemia–reperfusion
MSCs: Mesenchymal stem cells
BMSCs: Bone mesenchymal stem cells
Hypo-Exo: Hypoxia-preconditioned BMSCs-derived exosomes
Exo: BMSCs-derived exosomes
HUVECs: Human umbilical vein endothelial cells
OGD: Oxygen glucose deprivation
ROG: Reoxygenation and reglucose
ROS: Reactive oxygen species
GO: Gene ontology
KEGG: Kyoto Encyclopedia of Genes and Genomes
